# C-reactive protein concentration in bipolar disorder: association with genetic variants

**DOI:** 10.1186/s40345-019-0162-z

**Published:** 2019-12-02

**Authors:** Ann-Kristin Evers, Julia Veeh, Rhiannon McNeill, Andreas Reif, Sarah Kittel-Schneider

**Affiliations:** 1 Department of Psychiatry, Psychosomatic Medicine and Psychotherapy, University Hospital Frankfurt, Goethe University, Frankfurt Am Main, Germany; 2 Department of Psychiatry, Psychosomatic Medicine and Psychotherapy, University Hospital, University of Würzburg, Margarete-Höppel-Platz 1, 97080 Würzburg, Germany

**Keywords:** Bipolar disorder, Genotype, C-reactive protein, Biomarker, Inflammation

## Abstract

**Background:**

Several recent studies have investigated the role of C-reactive protein (CRP) in bipolar disorder (BD), but few studies have directly investigated the interaction between *CRP* genetic variants and peripheral CRP concentration across different phases of BD. In this study, we aimed to replicate previous findings that demonstrated altered CRP levels in BD, and to investigate whether there is an association of peripheral protein expression with genetic variants in the *CRP* gene.

**Methods:**

221 patients were included in the study, of which 183 (all episodes, 46 not medicated, 174 medicated) were genotyped for *CRP* single-nucleotide polymorphisms (SNPs) shown to influence peripheral CRP protein expression (rs1800947, rs2808630, rs1417938, rs1205).

**Results:**

There were no differences in CRP levels associated with the genotypes, only regarding the rs1205 SNP there were significantly different CRP protein expression between the genotypes when taking body mass index, age, BD polarity, subtype and leukocyte number into account. However, we could show significantly elevated CRP protein expression in manic patients compared to euthymic and depressed patients, independent from genotype. Medication was found to have no effect on CRP protein expression.

**Conclusions:**

These results indicate that low grade inflammation might play a role in mania and might be rather a state than a trait marker of bipolar disorder.

## Background

Bipolar disorder (BD) is primarily a periodic disease that has a global burden (Ferrari et al. 2016). It is characterized by the occurrence of (hypo-)manic and depressive episodes, and in a number of patients these mood states increase in frequency and severity over time, as well as decreasing responsiveness to treatment and that can result in a chronic course (Gildengers et al. 2014). The pathogenesis of BD is still relatively unknown. According to current findings, it is a multifactorial disease (Pitchot et al. 2012). BD is among the psychiatric disorders with the highest heritability, which is estimated at about 80% (Craddock and Jones 1999). However, developmental and environmental risk factors are additionally thought to play a role in the pathogenesis of the disorder (Landgraf et al. 2014).

There is increasing evidence that neuro-inflammation may play a role in the molecular pathomechanisms of BD (Muneer 2016). Increased levels of the inflammatory marker C-reactive protein (CRP) have been reported in BD in comparison to healthy controls (Bai et al. 2014; Chang and Chen 2017; Dargél et al. 2015). Elevated CRP levels have also been associated with an increased level of all-cause mortality in BD (Hayes et al. 2015). BD has been linked to metabolic and immunologic dysregulations (Berk et al. 2013; Leboyer et al. 2016; Modabbernia et al. 2013; Munkholm et al. 2013), and in BD patients, there appears to be a high comorbidity with somatic diseases such as cardiovascular disease, diabetes mellitus and autoimmune thyroiditis (Benros et al. 2013; Chakrabarti 2011; Rosenblat et al. 2017). In support of this, mortality due to cardiovascular disease has been reported as doubled in BD patients (Osby et al. 2001). Taken together, it has therefore been suggested that there are inflammatory subgroups in BD, and possibly also in other psychiatric disorders (Osimo et al. 2018). This would place these types of mood disorder in an inflammatory disease cluster, together with e.g. cardiovascular disease and obesity, in a “diseasesome” network (Midic et al. 2009; Perrino et al. 2017). Delineating this cluster, for example with the help of biomarkers, might pinpoint a group of patients which could be responsive to treatment options targeting the inflammasome.

The potential role and the direction of causality of CRP in the pathogenesis of BD is currently unclear. It has been hypothesized that stress can trigger an increase in CRP expression, which in turn leads to increased permeability of the blood–brain barrier. This consequently permits easier diffusion across the barrier for certain molecules, such as pro-inflammatory cytokines or auto-antibodies, which could cause multiple abnormalities in the brain (Hsuchou et al. 2012). CRP levels may act as a mood state marker in BD (Dargél et al. 2015; Jacoby et al. 2016; Wysokinski et al. 2015), and high levels of CRP may also represent an early warning sign for the onset of manic symptoms in depressed patients (Becking et al. 2013). Studies in the general population suggest that elevated CRP levels may even be predictive for the onset of BD (Wium-Andersen et al. 2016), and high levels of CRP are significantly correlated with BD disease severity (Dickerson et al. 2007; Lee et al. 2013). However, there are also negative findings regarding an association of CRP and BD, which may be partly due to the immunosuppressive actions of mood stabilizing medication (Dickerson et al. 2015; Haarman et al. 2014). Furthermore, it is not known what may trigger CRP production in BD patients. External stressors might activate the hypothalamus–pituitary–adrenal (HPA) axis, leading to increased secretion of cortisol and adrenergic hormones and an according change in immunological processes. However as elevated CRP levels are only found in a subgroup of BD patients, genetic susceptibility might pay a role. In support of this, several genetic polymorphisms in the CRP and other genes have been shown to influence peripheral CRP levels in different cohorts of psychiatric and non-psychiatric patients, as well as the general population (Carlson et al. 2005; Halder et al. 2010; Pankow et al. 2001; Prins et al. 2016; Reiner et al. 2012).

The first aim of the present cross-sectional study was to determine whether *CRP* genotype influences peripheral CRP concentration in BD patients. Patients were genotyped for the previously published functional *CRP* single nucleotide polymorphisms (SNPs) rs1800947 (Halder et al. 2010), rs1417938 (Henderson et al. 2015; Martínez-Calatrava et al. 2007), rs1205 (Halder et al. 2010; Flores-Alfaro et al. 2012) and rs2808630 (Kittel-Schneider et al. 2018). We then investigated a possible association of current polarity, bipolar subtype, medication and several other variables with serum CRP concentration.

## Methods

### Participants

A naturalistic sample of 221 BD patients in all mood phases (depressed, manic, mixed, euthymic) were recruited from 2009 to 2014 who were treated as inpatients at the Department of Psychiatry, Psychosomatic Medicine and Psychotherapy, University Hospital Würzburg, Würzburg, Germany. The patients were all of Caucasian origin and mostly from the lower Franconian area. The patients were diagnosed according to DSM-IV criteria independently by two specialists (SKS and AR). Information about smoking status, BMI, medication, CRP values, renal function and leukocyte values was obtained from the clinical routine data during the inpatient treatment. Blood for CRP analysis was drawn of the majority of the patients when the patients were admitted, only in few cases (the euthymic cases) blood was taken for CRP levels when the patients were discharged. A detailed description of sample characteristics and demographic data is presented in Table 1. Inclusion criteria were a diagnosis of bipolar disorder as primary psychiatric diagnosis and age ≥ 18 years. Exclusion criteria were severe somatic disorders (severe cardiovascular disease, severe neurological diseases, acute or chronic infections, carcinomas and autoimmune diseases) and organic affective disorders. Written informed consent was obtained from all participants before study evaluation. The authors assert that all procedures contributing to this work comply with the ethical standards of the relevant national and institutional committees on human experimentation and with the Helsinki Declaration of 1975, as revised in 2008. The ethics committee of the University of Würzburg approved the study.

**Table 1:** Sample characteristics and demographic data of bipolar patients

	n	Ratio
Sex (male/female)	86/135	0.64
Cigarettes (smoker/non-smoker)	100/103	0.97

	n	M	SD
Age at sampling (years)	221	49.6	15.10
BMI (kg/m^2^)	139	28.2	6.01
Leukocytes (1000/μl)	204	6.6	3.77
MDRD (ml/min/1.73 m^2^)	206	86.7	23.58

Medication	N
Lithium only MS	9
Anticonvulsant only MS	5
Antipsychotic only MS	16
Combination Li + antidepressant	14
Combination Li + antipsychotic	15
Combination Li + antidepressant + antipsychotic	28
Combination Li + anticonvulsant + antidepressant	6
Combination Li + anticonvulsant + antipsychotic	8
Combination Li + anticonvulsant + antidepressant + antipsychotic	6
Combination anticonvulsant + antidepressant	9
Combination anticonvulsant + antipsychotic	14
Combination antidepressant + antipsychotic	31
Combination anticonvulsant + antidepressant + antipschotic	14
No mood stabilizer	46
Only antidepressants	16
No psychotropic medication	30
Medication not available	1

Subtype	N
Bipolar I/Bipolar II/Bipolar III/not specified	108/85/5/23

Polarity at sampling	N
Euthymic	7
Depressed	123
Manic (including hypomanic and mixed states)	89
Illness duration (in years)	18.14 ± 12.5
Number of depressed episodes	9.67 ± 18.55
Number of manic episodes	1.75 ± 7.97
Number mixed episodes	7.06 ± 16.85
Number of all previous episodes	17.51 ± 35.91

*BMI* body mass index, *MDRD* Modification of Diet in Renal Disease, *MS* mood stabilizer, *Li* lithium

### Genotyping

183/221 participants gave approval for genetic testing. DNA was isolated from ethylenediaminetetraacetic acid (EDTA)-monovettes using the de-salting method (Miller et al. 1988) and DNA concentration and quality was assessed by spectrophotometric measurement (Infinite^®^ 200 PRO, *Tecan Group Ltd., Männedorf, Switzerland*). Competitive Allele Specific Polymerase Chain Reaction (KASP) assay (He et al. 2014) was performed according to manufacturer’s instructions (LGC Genomics, *Berlin*, *Germany*) to genotype previously published SNPs in the *CRP* gene (rs1800947, rs1417938, rs1205 and one tagSNP rs2808630). The SNPs were selected due to previously published studies in different study population which showed that those common variants might influence the CRP protein levels [rs1800947 (Halder et al. 2010), rs1417938 (Henderson et al. 2015; Martínez-Calatrava et al. 2007), rs1205 (Halder et al. 2010; Flores-Alfaro et al. 2012) and rs2808630 (Kittel-Schneider et al. 2018)].

5 to 50 ng/µl of DNA was tested in a 384-well plate by Wet DNA method. Fluorescent signal from the two FRET (fluorescence resonance energy transfer) cassettes [frequency- and amplitude modulation (FAM) and hexachlorocyclopentadiene (HEX)] was detected with a LightCycler^®^ 480 (F. Hoffmann-La Roche Ltd., *Basel, Switzerland*). In order to confirm the genotyping quality, we analyzed samples in duplicates as internal controls on each plate, including negative water controls. As an external control, two additional samples whose genotypes were already known were also included on each plate, confirming the genotyping quality over all measurements. Genotype frequencies of all tested *CRP* SNPs were consistent with the Hardy–Weinberg equilibrium. Recently, rs17460165, rs17860481 and rs52802864 SNPs have been merged into one SNP, rs1800947. However, C and G alleles are the most common alleles in the European population, therefore it did not appear necessary to genotype for other newly described alleles (see http://www.ensembl.org/Homo_sapiens/Variation/Sequence?db=core;r=1:159713148-159714148;v=rs1800947;vdb=variation;vf=1229971).

### CRP serum levels

CRP serum levels were measured in routine blood parameter checks when patients were hospitalized. Serum CRP was measured with the cobas^®^ system (cobas^®^ Integra 800, cobas^®^ 6000, cobas^®^ 8000, *Roche Diagnostics*, *Indianapolis*, *USA*) according to manufacturer’s instructions. This system measures human CRP by agglutination with latex particles which are coated with anti-CRP-antibodies. The amount of agglutination is then measured by turbidimetry at 552 nm. The normal range of CRP with this method is given between 0.0 and 0.5 mg/dl.

### Statistical analysis

Statistical analyses were conducted with SPSS (Statistical Package for the Social Sciences for Windows, version 24, IBM Corp., Armonk, New York). CRP levels were not normally distributed, as revealed by testing with the Kolmogorov–Smirnov and Shapiro–Wilk tests (both p < 0.001). Therefore non-parametric tests were conducted to determine whether genotype differences could be seen in CRP expression (Kruskal–Wallis test, Mann–Whitney U-test). When the number of patients was large enough in the subgroup analysis, for example regarding the episodes, ANOVA and ANCOVA were also performed. For those SNPs whose subgroups were large enough, we additionally conducted exploratory ANOVA and ANCOVA analysis including several covariates. Correlation of CRP with metric variables was tested by Spearman’s test. The level of significance was corrected for multiple comparison with Bonferroni correction (4 tested SNPs for the main hypothesis, p ≤ 0.05/4) and set at p ≤ 0.013.

## Results

### Association of confounders with CRP levels

CRP serum levels in the whole sample were significantly positively correlated with body mass index (Spearman’s correlation *r *= 0.359, *p *< 0.0001), age (Spearman’ s correlation *r *= 0.186, *p *= 0.005) and leukocyte numbers (Spearman’s correlation, *r* = 0.253, *p *< 0.0001). Renal function as measured by Modification of Diet in Renal Disease (MDRD) value were not significantly correlated with CRP levels. Even though nominally increased CRP values were observed in male patients (*n *= 86, *M *= 0.4280 mg/dl, *SD* 0.90729) compared to female patients (*n *= 135, *M *= 0.3805 mg/dl, *SD* 0.05137), the difference was not statistically significant [*t* (219) = − 0.470, *p *= 0.639]. The same applies to the nominally increased CRP for smokers (*n *= 100, *M *= 0.446 mg/dl, *SD* 0.85207) compared to non-smokers (*n *= 103, *M *= 0.3670 mg/dl, *SD* 0.65085). CRP levels were not significantly different between patients taking lithium and patients taking other medication (lithium group n = 86 vs. non-lithium group n = 135, Mann–Whitney-U-test, *p *= 0.512).

**Fig. 1:**
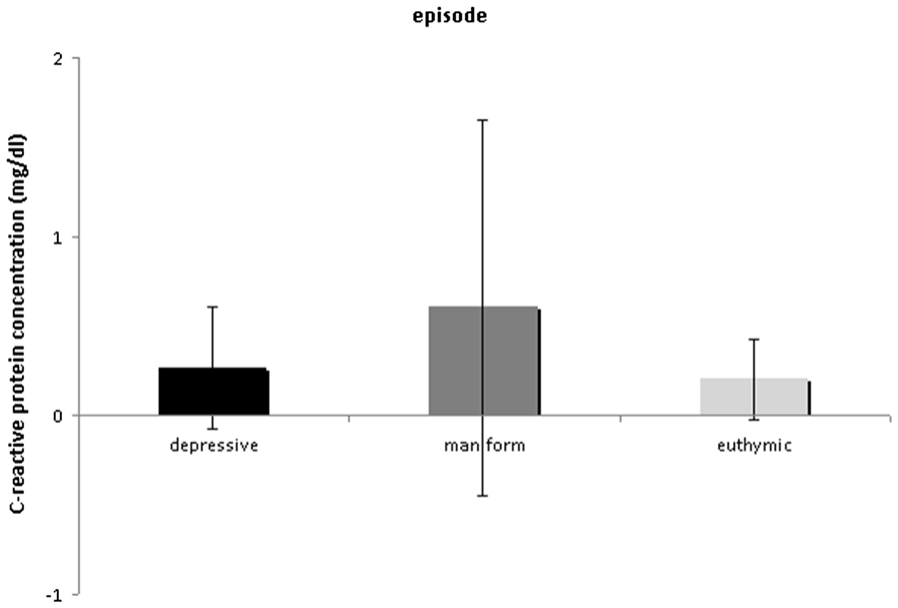
Measured levels of C-reactive protein levels in bipolar patients in different episodes. Manic patients (n = 89) showed significantly higher C-reactive protein levels as compared to euthymic (n = 7) and depressed patients (n = 123) (ANOVA, *p *= 0.003). C-reactive protein levels are given as mean values ± standard deviation

The BD group as a whole had a mean CRP concentration of 0.40 ml/d*l* (± 0.73 mg/dl SD; 95% CI 0.30 to 0.50), with significantly elevated CRP levels during manic episodes after correction for multiple comparison [ANOVA, *F* (2, 216) = 5.888, *p *= 0.003] (see Fig. 1) compared to euthymic and depressed episodes. Manic patients had a mean CRP level concentration of 0.60 mg/dl (± 1.05 mg/dl *SD*, with a range between 0.01 mg/dl and 5.90 mg/dl), while euthymic patients had a mean concentration of 0.20 mg/dl (± 0.22 mg/dl *SD*, with a range between 0.03 mg/dl and 0.68 mg/dl) and depressed patients had a mean concentration of 0.27 mg/dl (± 0.34 mg/dl *SD*, with a range between 0.01 mg/dl and 1.99 mg/dl) (see Fig. 1). Significant differences were additionally found relating to the subtypes of BD. Patients classified as bipolar subtype I (*n *= 108, *M *= 0.5340 mg/dl, *SD* 0.07176) had significantly elevated CRP levels [*t* (139, 792) = 2.717, *p *= 0.007] in comparison to bipolar subtype II (*n *= 85, *M *= 0.2601 mg/dl, *SD* 0.34693), BD III and BD NOS. However, because there were significantly more manic episode patients in the BD I group (Chi-Square test, *p *= 0.005), an exploratory ANCOVA was calculated including episode as a covariate. The result was then no longer significant after correction for multiple comparison (ANCOVA, CRP concentration differences between BD I, BD II, BD III and BD NOS, *p *= 0.032). Duration of illness was significantly positively correlated with CRP levels (Spearman’s correlation *r *= 0.212, *p *= 0.004).

### Association of genetic variation in the CRP gene with serum CRP concentration

We assessed if there were differences between the genotypes of four SNPs (rs1800947, rs1417938, rs1205 and rs2808630) in CRP levels (Table 2). No significant differences in CRP levels were detected between different genotypes using non parametric Kruskal–Wallis analysis. However, exploratory ANCOVA analysis using body mass index, leukocyte number, age, current polarity and bipolar subtype as covariables revealed significant differences between the genotypes in the rs1205 SNP, which remained marginally significant even after correcting for multiple comparison [ANCOVA, *F* (2, 710) = 1.023, *p *= 0.013]. No significant differences were found for any of the other SNPs. However, there was a trend for increased CRP expression in the minor AA carriers of the rs1417938 SNP, for decreased CRP levels in the minor allele carriers of rs2808630 and rs1800947 SNPs (Table 2). The distribution of all genotypes, call rates and minor allel frequency is given in Additional file 1: Table S1.

**Table 2:** Genotype distribution and C-reactive protein concentration in bipolar patients

rs1800947	GG	CG	CC
n	140	17	2
CRP [mg/dl]	0.39 ± 0.73 SD	0.19 ± 0.23 SD	0.25 ± 0.10 SD

rs1417938	TT	AT	AA
n	89	75	15
CRP [mg/dl]	0.42 ± 0.88 SD	0.36 ± 0.52 SD	0.32 ± 0.27 SD

rs1205	CC	CT	TT
n	87	69	25
CRP [mg/dl]	0.41 ± 0.71 SD	0.32 ± 0.44 SD	0.43 ± 1.18 SD

rs2808630	TT	CT	CC
n	83	80	10
CRP [mg/dl]	0.37 ± 0.71 SD	0.35 ± 0.54 SD	0.82 ± 1.55 SD

Genotype distribution of the 4 investigated SNPs in the whole sample is displayed as well as mean CRP concentration ± SD

*CRP* C-reactive protein, *n* number, *SD* standard deviation

Additionally, we evaluated if there was a different distribution of the genotypes between the different current episodes, which was not the case (Chi Square-test, every *p* ≥ 0.5, see Additional file 2: Table S2a–d).

## Discussion

We were able to replicate previous findings of increased CRP levels in BD patients suffering from a manic episode as compared to euthymic and depressed patients (Horsdal et al. 2017; Wysokinski et al. 2015). This finding is also supported by a previous meta-analysis (Fernandes et al. 2016). However, inconsistent findings have been reported, finding either no CRP level differences between episodes or also elevated CRP levels between in depressed vs. euthymic patients (Balukova et al. 2016; Jacoby et al. 2016; Tsai et al. 2014). Furthermore, a previous meta-analysis has shown significantly elevated CRP levels in manic but also in euthymic patients compared to depressed patients with BD (Dargél et al. 2015). Taken together, those results point to an inflammatory component in BD and the results of our own and previous studies suggest that elevated CRP levels might rather be a state than a trait marker of BD (Dargél et al. 2015). However, it has to be considered, that most studies are investigating medicated patients and medication might have an effect on CRP concentration. But it has been published that increased CRP levels could be measured during the manic episode of BD I patients, in this sample only in medication-free patients (Uyanik et al. 2015). Even though the anti-inflammatory effects of lithium could be demonstrated in earlier studies (Beurel and Jope 2014; Fond et al. 2014), lithium (among other drugs) did not have a significant effect on CRP concentration in our study which is consistent with a previous meta-analysis (Dargél et al. 2015).

As already shown, we also found an association between CRP level and body mass index (Bai et al. 2015; Marshe et al. 2017), and between CRP levels and patient’s age (Avramopoulos et al. 2015; Wysokinski et al. 2015). Contrary to Horsdal et al. (2017), we were able to confirm a correlation between raised CRP levels and the white blood cell count, as documented in an earlier study (Tsai et al. 2014). However, we found only nominal but no significantly elevated CRP in the smokers vs. non-smokers in our study, in previous studies smoking significantly influenced CRP levels (Bai et al. 2015; Huang et al. 2013).

Although we could find an association between CRP concentration and mood episode, the directionality of this association cannot be positively inferred. Thus, the links between inflammation and this psychiatric disorder remain unclear and may be bi-directional. Further studies should investigate the relationship between CRP concentration and the progression of BD, and BD’s potential role as an epiphenomenon of low-grade inflammation. Another explanation might be that increased CRP is due to activation of the stress axis in manic episodes because of the general increase in activity (psychomotor activity, lack of sleep, etc.). Additionally, we found a significant correlation of increased CRP levels with longer duration of the illness which could be interpreted as a sign of neuronal progression of the course of BD over the life span.

Regarding the question if CRP levels could be used as biomarker for early detection of emerging episodes in bipolar disorder or a prognostic biomarker for the course of the disorder of the life span including the development of somatic comorbidities, there are several issues that need to be addressed in future studies. The CRP level differences in the manic patients compared to the euthymic as well in our study as in previous studies are very small even if they are significantly different (Dargél et al. 2015; Uyanik et al. 2015). Therefore it is difficult to define a cut-off CRP level that could be used as an early detection for emerging manic episodes in the individual patient as well as use this as a prognostic marker for a more severe course or development of comorbid somatic diseases. It might prove more fruitful to develop composite-biomarkers for personalized early detection or prognosis by the integration of CRP and potentially other protein markers and genetic, imaging and neuropsychological data. However, to define inflammatory subgroups that might benefit from add-on inflammatory therapeutics might be possible using only slightly elevated CRP levels (Müller 2019; Soczynska et al. 2009).

In our study, there were hints that there are differences between the rs1205 SNP genotypes in our sample of bipolar patients regarding CRP levels. In a recent study investigating late life depression, female rs1205 TT carriers displayed significantly more depressed symptoms but had lower CRP levels than the other genotypes, while the AA carriers of female rs1417938 were associated with a decreased risk of depression and had slightly elevated CRP levels (Ancelin et al. 2015). In contrast to our findings, a lower concentration of CRP was reported in men with greater odds of having clinically significant depression, but only in patients with the rs1205 minor AA genotype (Almeida et al. 2009). Similarly, the major allele CC of the rs1205 SNP was strongly associated with adolescent emotional problems and metabolic syndrome (Gaysina et al. 2011).

Minor rs1205 allele (TT) carriers, who showed in our study featuring significantly increased CRP levels, might also be at elevated risk for somatic disorders, especially including cardiovascular and metabolic diseases. Homozygous variant-type (TT) carriers of rs1205 had a threefold increased risk for heart valve reoperation and elevated CRP levels compared to ancestral allele carriers (Lee et al. 2016; Wypasek et al. 2015). Peric and collaborators also reported a trend toward higher maximum transvalvular gradient in carriers of T alleles in the rs1205 SNP, suggesting that the T allele could be a potential marker concerning severe and heavily calcified aortic stenosis (Peric et al. 2013). The susceptibility and severity of community-acquired pneumonia was additionally associated with the frequency of the *CRP* rs1205 TT genotype (Chou et al. 2016). In the future, follow-up studies would be of high interest to investigate if those BD patients of our sample with CRP risk variants and/or elevated CRP might be at an increased risk for cardiovascular disease.

The remarkable overlap between psycho-immuno-endocrinologic mechanisms in BD and cardiometabolic illnesses, as well as other chronic inflammatory diseases, may be due to common genetic variants. It is of note that the rare recessive genotype rs1800947 CC of the CRP gene was recently found to have a significant influence on CRP concentration, mortality and depressive symptoms in chronic heart failure patients; the highest risk CC carriers had the highest hs-CRP levels (Kittel-Schneider et al. 2018). In comparison, our study revealed a trend of decreased CRP levels for rs1800947 CC carriers in BD patients which is in line with previous findings in the Health 2000 Study (Kettunen et al. 2011) as well as in other previous studies (Carlson et al. 2005; Miller et al. 2005; Zee and Ridker 2002). The reason for the contrasting results is unclear and indicated that genetic influences might lead to differential effects in different disorders.

## Conclusions

In summary, we could add to the evidence that a part of the variance of CRP expression in serum could be explained by functional variants in the CRP gene. However, the direction and effect of those genetic variants seem to vary in different somatic and psychiatric disorders, as well as in the healthy general population. However, the differences between CRP levels between BD patients in a manic episode vs. depressed and euthymic patients were much more pronounced than the genotype effect. Further studies are therefore needed to confirm these current findings, and to determine whether CRP might be indeed more useful as an early detection or prognostic marker in BD or at least used as a part of a composite-biomarker, or might be suitable as a biomarker for distinguishing inflammatory subgroups.

### Significant outcomes

CRP protein levels were significantly increased in bipolar manic patients.

BMI, age and leukocyte levels were positively correlated with CRP protein levels.

Duration of illness was positively correlated with CRP protein levels.

### Strengths and limitations

The results of our study have some limitations. Firstly, the peripheral CRP level within the blood may also be influenced by further external factors which we did not take into account. Not only may the covariates considered by the present study (such as smoking or body mass index) play a role, but other parameters such as physical activity, an unhealthy lifestyle or stress may also influence CRP concentration. We were unable to include these other possible covariates in this study. Secondly, even though we excluded patients from the study who demonstrably suffered from current infections or carcinomas, it is possible that undiagnosed illnesses may have affected CRP levels. Thirdly, longitudinal data from different time-points would have been more informative in the search for trait- or state biomarkers and inflammatory subtypes. Finally, the validity of the genetic results is limited by the relatively low number of patients.

The strength of the study is that we investigated a naturalistic sample of bipolar patients in all episodes and we could take several variables that might influence CRP levels into account because the information was available. Furthermore, this sample consisted of a relatively homogenous origin so that influences on CRP levels and differences in CRP genetic variants due to the origin of the patients are reduced.

## Supplementary information

**Additional file 1: Table S1.** Distribution of genotypes of all tested SNPs.

**Additional file 2: Table S2.** a. Distribution of rs2808630 genotypes with regard to current episode. b. Distribution of rs1417938 genotypes with regard to current episode. c. Distribution of rs1205 genotypes with regard to current episode. d Distribution of rs1800947 genotypes with regard to current episode.

## Data Availability

Data are available on request.

## Abbreviations

BD: bipolar disorder
CRP: C-reactive protein
SNPs: single nucleotide polymorphisms
MDD: major depressive disorder
BMI: body mass index
MDRD: Modification of Diet in Renal Disease
MS: mood stabilizer
Li: lithium
HPA axis: hypothalamus–pituitary–adrenal axis
KASP: Competitive Allele Specific Polymerase Chain Reaction
EDTA: ethylenediaminetetraacetic acid
FRET: fluorescence resonance energy transfer
FAM: frequency- and amplitude modulation
HEX: hexachlorocyclopentadiene
