# Supplementary material for: C-reactive protein concentration in bipolar disorder: association with genetic variants
Source: Int J Bipolar Disord. 2019 Dec 2;7:26. doi: 10.1186/s40345-019-0162-z (PMC6885457; doi:10.1186/s40345-019-0162-z)
Supplement: Supplementary file 1 — Additional file 1: Table S1. Distribution of genotypes of all tested SNPs. [file 40345_2019_162_MOESM1_ESM.docx]

**Table S1:** Distribution of genotypes of all tested SNPs

| **SNP** | **n** | **Major genotype** | **Heterozygous** | **Minor genotype** | **Call rate** | **Global Minor allele frequency (MAF)** |
| --- | --- | --- | --- | --- | --- | --- |
| *CRP* |  |  |  |  |  |  |
| rs1800947 | 159 | GG: 140 (88.1%) | CG: 17 (10.7%) | CC: 2 (1.3%) | 87 % | C: 3% |
| rs1417938 | 179 | TT: 89 (49.72%) | AT: 75 (41.9%) | AA: 15 (8.38%) | 97 % | A: 19.2% |
| rs1205 | 181 | CC: 87 (48.1%) | CT: 69 (38.12%) | TT: 25 (13.81%) | 99 % | T: 33.8% |
| rs2808630 | 173 | TT: 83 (48% ) | CT: 80 (46.24%) | CC: 10 (6%) | 95 % | C: 22.6% |

Global minor allele frequency taken from https://www.ncbi.nlm.nih.gov/snp/
