# Supplementary material for: C-reactive protein concentration in bipolar disorder: association with genetic variants
Source: Int J Bipolar Disord. 2019 Dec 2;7:26. doi: 10.1186/s40345-019-0162-z (PMC6885457; doi:10.1186/s40345-019-0162-z)
Supplement: Supplementary file 2 — Additional file 2: Table S2. a. Distribution of rs2808630 genotypes with regard to current episode. b. Distribution of rs1417938 genotypes with regard to current episode. c. Distribution of rs1205 genotypes with regard to current episode. d Distribution of rs1800947 genotypes with regard to current episode. [file 40345_2019_162_MOESM2_ESM.docx]

**Table S2a:** Distribution of rs2808630 genotypes with regard to current episode

| **rs2808630** | | | | | | |
| --- | --- | --- | --- | --- | --- | --- |
|  | | | | | | |
|  | | Episode | | | Total | p |
|  |  | depressive | manic | euthymic |  |  |
| rs2808630 | TT | 51 | 30 | 2 | 83 | 0.70 |
|  | CT | 41 | 35 | 3 | 79 |  |
|  | CC | 5 | 5 | 0 | 10 |  |
| Total | | 97 | 70 | 5 | 172 |  |

Distribution of genotypes between current episodes were calculated by χ²-test.

**Table S2b:** Distribution of rs1417938 genotypes with regard to current episode

| **rs1417938** | | | | | | |
| --- | --- | --- | --- | --- | --- | --- |
|  | | | | | | |
|  | | Episode | | | Total | p |
|  |  | depressive | manic | euthymic |  |  |
| rs1417938 | TT | 51 | 35 | 2 | 88 | 0.88 |
|  | AT | 41 | 31 | 3 | 75 |  |
|  | AA | 9 | 5 | 1 | 15 |  |
| Total | | 101 | 71 | 6 | 178 |  |

Distribution of genotypes between current episodes were calculated by χ²-test.

**Table S2c:** Distribution of rs1205 genotypes with regard to current episode

| **rs1205** | | | | | | |
| --- | --- | --- | --- | --- | --- | --- |
|  | | | | | | |
|  | | Episode | | | Total | p |
|  |  | depressive | manic | euthymic |  |  |
| rs1205 | CC | 48 | 34 | 4 | 86 | 0.83 |
|  | CT | 38 | 29 | 1 | 68 |  |
|  | TT | 15 | 9 | 1 | 25 |  |
| Total | | 101 | 72 | 6 | 179 |  |

Distribution of genotypes between current episodes were calculated by χ²-test.

**Table S2d:** Distribution of rs1800947 genotypes with regard to current episode

| **rs1800947** | | | | | | |
| --- | --- | --- | --- | --- | --- | --- |
|  | | | | | | |
|  | | Episode | | | Total | p |
|  |  | depressive | manic | euthymic |  |  |
| rs1800947 | GG | 81 | 53 | 5 | 139 | 0.51 |
|  | GC | 8 | 9 | 0 | 17 |  |
|  | CC | 2 | 0 | 0 | 2 |  |
| Total | | 91 | 62 | 5 | 158 |  |

Distribution of genotypes between current episodes were calculated by χ²-test.
